# Short-Term Memory Dynamics of TiN/Ti/TiO_2_/SiO*_x_*/Si Resistive Random Access Memory

**DOI:** 10.3390/nano10091821

**Published:** 2020-09-12

**Authors:** Hyojong Cho, Sungjun Kim

**Affiliations:** Division of Electronics and Electrical Engineering, Dongguk University, Seoul 04620, Korea; chj9102@dgu.ac.kr

**Keywords:** memristor, synapse device, neuromorphic computing, short-term memory, titanium dioxide

## Abstract

In this study, we investigated the synaptic functions of TiN/Ti/TiO_2_/SiO*_x_*/Si resistive random access memory for a neuromorphic computing system that can act as a substitute for the von-Neumann computing architecture. To process the data efficiently, it is necessary to coordinate the information that needs to be processed with short-term memory. In neural networks, short-term memory can play the role of retaining the response on temporary spikes for information filtering. In this study, the proposed complementary metal-oxide-semiconductor (CMOS)-compatible synaptic device mimics the potentiation and depression with varying pulse conditions similar to biological synapses in the nervous system. Short-term memory dynamics are demonstrated through pulse modulation at a set pulse voltage of −3.5 V and pulse width of 10 ms and paired-pulsed facilitation. Moreover, spike-timing-dependent plasticity with the change in synaptic weight is performed by the time difference between the pre- and postsynaptic neurons. The SiO*_x_* layer as a tunnel barrier on a Si substrate provides highly nonlinear current-voltage (I–V) characteristics in a low-resistance state, which is suitable for high-density synapse arrays. The results herein presented confirm the viability of implementing a CMOS-compatible neuromorphic chip.

## 1. Introduction

Von-Neumann computing systems, in which a central processing unit reads data in memory and processes information, constitute the dominant architecture of modern general-purpose computers. The disadvantage of this architecture is that it leads to a bottleneck between the memory and the central processing unit when managing large amounts of data. Lags in data processing can present challenges in applications such as in artificial intelligence (AI) and the Internet of Things (IoT), where massive data are required to be processed in the short term. Hence, the development of novel efficient computing systems is essential for handling massive data [1,2]. A novel data processing system that mimics the human brain was reported in various research studies. Currently, research is underway on how to utilize such a system to solve problems in a similar way to the human brain [3,4]. A brain composed of 10^11^ neurons and 10^15^ synapses can swiftly perform high-dimensional functions such as learning and judgment while consuming only about 20 W per hour. This consumption is much smaller than that of a conventional computing system, which consumes approximately 56 kW per hour [5,6,7,8]. A neuromorphic system can emulate biological synapses on a hardware level, with the aim of a low power consumption, fault tolerance, and high efficiency processing [9,10,11]. By structuring integrated circuits in the form of artificial neural networks, it is possible to process data for each neural network. Likewise, by reducing data movement between memory and central processing units and enabling local data management, processing is more efficient and bottlenecks can be minimized. Similar to the neurobiological architecture in the human brain, neuromorphic systems have artificial neurons acting as computing elements and synapses acting as memory elements. Resistive random access memory (RRAM) is being explored as one of the candidates [12,13,14,15,16,17,18,19,20] to replicate the characteristics of a biological synapse. It has an advantage over phase-change memory [21] and ferroelectric memory [22], i.e., it has a low power consumption [23,24,25,26]. RRAM has additional advantages, such as a high density [23] and fast switching speed [24], which can be obtained from a two-terminal structure for neuromorphic systems. When a stimulus, that is, pulse or DC voltage, is applied to an oxide-based RRAM, conducting defects (oxygen vacancies) are formed in the insulating layer [27,28].

As shown in numerous previous studies, RRAM has been extensively reported to mimic synaptic characteristics [29,30,31,32,33] as well as to implement nonvolatile high-density memory [34]. Anion migration and metal ion migration are representative RRAM operation systems [32]. Sudden changes in conductance with high current for filamentary switching RRAM have a stochastic nature when the device changes from a high-resistance state (HRS) to a low-resistance state (LRS), which presents a major limitation of synaptic devices in neuromorphic computing [35,36]. This is because abrupt switching is difficult to implement in many conductance states [37]. As an improvement to this problem, nonfilamentary switching, which exhibits the characteristics of gradual changes in switching, is preferred for the purpose of synaptic devices. In addition, data loss over time in nonfilamentary switching can be utilized as short-term memory (STM) and reservoir computing with temporal processing, as demonstrated in previous studies [38].

In this study, we present a TiN/Ti/TiO_2_/SiO*_x_*/Si multilayer structured device that can mimic synapse characteristics. In the past, several TiO_2_-based synaptic devices with nonfilamentary switching were reported [39,40], but studies considering short-term memory effects are scarce in the literature. Moreover, the Si substrate as the bottom electrode (BE) in our device has several advantages for RRAM applications. The self-rectifying characteristics are achieved by varying the concentration of impurities on the Si surface [41]. In addition, the Si surface can be scaled by anisotropic wet etching, which can improve switching performances [42]. The SiO*_x_* as a tunnel barrier layer can be easily grown during the subsequent process [43]. The nonlinearity and dynamic range of potentiation and depression were investigated by controlling the pulse width and pulse voltage. Furthermore, STM and paired-pulse facilitation (PPF) are demonstrated by adjusting the pulse interval time. Finally, the spike-timing-dependent plasticity (STDP)-like curve was achieved spike-timing-dependent-plasticity by designing the pre- and postspikes.

## 2. Materials and Methods

The proposed TiN/Ti/TiO_2_/SiO*_x_*/Si device was fabricated as follows. First, a 200 nm thick, highly doped n-type Si BE was deposited through a low-pressure chemical vapor deposition (LPCVD) by reacting SiH_4_ and PH_3_ on the SiO_2_/Si substrate. Then, a 13 nm thick TiO_2_ film was deposited via a DC sputter at room temperature. The flow rates of argon and oxygen were 12 and 8 sccm, respectively. For the TiO_2_ film deposition, a working pressure of 1 mTorr, power of 0.5 kW, and frequency of 50 kHz were applied. A 10 nm thick Ti top electrode was deposited on the TiO_2_ layer via a 100 μm diameter shadow mask under a working pressure of 5 mTorr, DC power of 5 kW, and argon flow rate of 50 sccm. Finally, to avoid the oxidation of Ti, additional nitrogen gas at a flow rate of 50 sccm was injected during 100 nm thick TiN deposition on the Ti top electrode. The electrical properties in the DC sweep and transient modes were measured using a semiconductor parameter analyzer (Keithley 4200-SCS and 4225-PMU ultrafast module, Solon, OH, USA).Transmission electron microscope (TEM) and energy-dispersive X-ray spectroscopy (EDS) was conducted by the JEOL (JEM-2100F, Tokyo, JAPAN). During the measurements, the bias voltage and pulse were applied to the Ti/TiN top electrode while the doped-poly Si bottom electrode was grounded.

## 3. Results and Discussion

**Figure 1:**
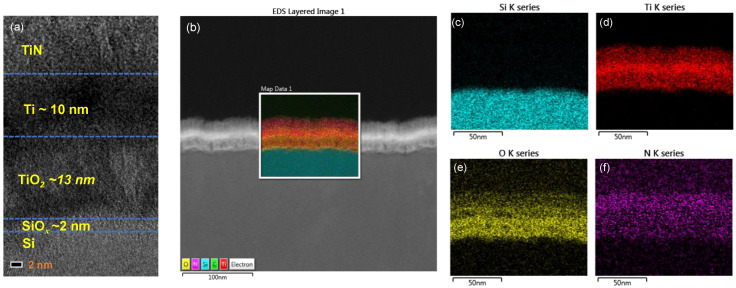
(**a**) TEM image TiN/Ti/TiO_2_/SiO*_x_*/Si device; (**b**) energy-dispersive X-ray spectroscopy (EDS) layered image in scanning transmission electron microscope (STEM); each element ((**c**) Si, (**d**) Ti, (**e**) O, (**f**) N) of TiN/Ti/TiO_2_/SiO*_x_*/Si device.

Figure 1a shows the cross-sectional TEM image of the TiN/Ti/TiO_2_/SiO*_x_*/Si device, where 2 nm thick SiO*_x_* and 13 nm thick TiO_2_ films can be distinguished. The SiO*_x_* layer is a native oxide that is inevitable during the process and can provide positive effects on resistive switching by acting as a tunnel oxide. To avoid further oxidation, TiN was deposited on the Ti top electrode. Figure 1b shows the EDS layers in a scanning TEM image for the TiN/Ti/TiO_2_/SiO*_x_*/Si stack. Each element (Si, Ti, O, and N) is displayed for each layer (Figure 1c–f), respectively. 

**Figure 2:**
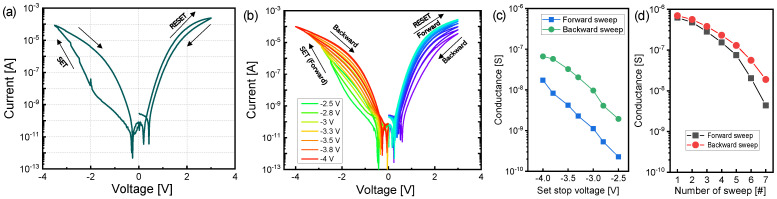
Current-voltage (I–V) curves and multilevel conductance characteristics of TiN/Ti/TiO_2_/SiO*_x_*/Si device: (**a**) typical I–V curves; (**b**) current change characteristics by repeated sweep from −2.5 to −4 V for set process and fixed 3 V for reset process; (**c**) conductance gradually increases with incremental set stop voltage; (**d**) conductance gradually decreases using the same voltage sweep (3 V).

Figure 2a shows typical current-voltage (I–V) curves of the TiN/Ti/TiO_2_/SiO*_x_*/Si device. Gradual resistive switching from the stimulus of the DC voltage sweep occurred without a forming operation. Nonfilamentary switching has the advantage of the operating current decreasing as the area of the device decreases [44]. Therefore, even in the TiN/Ti/TiO_2_/SiO*_x_*/Si device, a lower current could flow at a smaller cell size. The HRS changes to an LRS while sweeping the negative bias without the compliance current. A resistive switching operation that is applied without compliance to a device has the advantage of reducing circuit components that limit current. By applying a positive bias, the reset process prompts the device from the LRS to the HRS. The TiN/Ti/TiO_2_/SiO*_x_*/Si device in the LRS shows a rectifying property—the current is suppressed in the negative region compared to that of the positive region. The rectifying property can enlarge the array size in the cross-point structure by reducing the sneak current paths. The cycle-to-cycle variation of the LRS and HRS are presented in Figure S1.

Next, we demonstrate multilevel states of the TiN/Ti/TiO_2_/SiO*_x_*/Si device under the DC sweep mode. Multilevel conductance modulation is crucial in implementing neuromorphic systems, e.g., by adjusting the weight at the synapse. Figure 2b shows the I–V characteristics by a repeated sweep. By increasing the set stop voltage from −2.5 to −4 V, the conductance increases by approximately 76 times and 34 times for forward and backward sweeps, respectively (Figure 2c). For reset operation, a sweep up to 3 V is repeated seven times. As a result, a gradual reduction in conductance was observed (Figure 2d). Here, the conductance values are extracted at −1 and 1 V for the set and reset processes, respectively.

**Figure 3:**
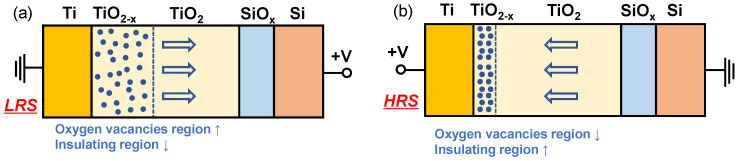
Illustration of a simple oxygen vacancy model to explain the conductance change of Ti/TiO_2_/SiO*_x_*/Si device in (**a**) low-resistance state (LRS) and (**b**) high-resistance state (HRS). Oxygen vacancies are full circles with blue color and the arrows indicate the moving direction of oxygen ions.

Next, we present the change in conductance in the TiN/Ti/TiO_2_/SiO*_x_*/Si device by an illustration that includes a simple oxygen vacancy configuration. The gradual conductance modulation in the TiO_2_-based RRAM system can be explained by the nonfilamentary switching model [39,40,45,46,47]. Resistive switching in the interface-type model occurs by barrier modulation at the interface between the electrode and insulator rather than by the rapid conductance change caused by the formation and rupture of local filaments [39,40,45,46,47]. Strong oxygen vacancies can be created at the interface between Ti and TiO_2_ because Ti is highly reactive to oxygen [45,46]. The oxygen vacancy region (defect region) became wider when a negative bias was applied to the top electrode (TiN/Ti), indicating that the insulating region (TiO_2_ layer, defect-less region) is reduced and then the conductance is increased for an LRS (Figure 3a). Conversely, the defect-free region is reduced when a positive bias is applied to the top electrode (TiN/Ti) for a HRS (Figure 3b).

**Figure 4:**
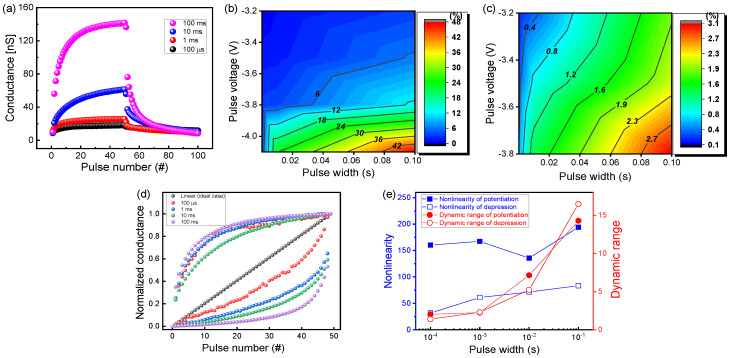
Potentiation and depression characteristics of TiN/Ti/TiO_2_/SiO*_x_*/Si device: (**a**) Pulse-width-controlled conductance change; contour maps of (**b**) potentiation and (**c**) depression as a function of pulse voltage and width; (**d**) normalized conductance in different pulse widths; (**e**) nonlinearity and dynamic range as a function of pulse width.

Next, we studied synaptic properties by pulse responses for the TiN/Ti/TiO_2_/SiO*_x_*/Si device. The amount of change in conductance (dynamic range) and linear weight update in a synaptic device are crucial factors for the implementation of hardware-based neuromorphic systems. Figure 4a shows the potentiation and depression curves at a fixed pulse voltage (−4 and 3.5 V for set and reset, respectively) while varying the pulse width from 100 μs to 100 ms. A read voltage of 0.5 V was used to convert conductance from the measured current after each set or reset pulse for 50 responses. A larger conductance change was observed for a larger pulse width. The change was more significant at the beginning of the pulse. The larger the pulse width, the longer the stimulus time applied to the device, thereby increasing the synaptic dynamic range. Figure 4b,c show the potentiation and depression contour mapping plots for the rate of change in conductance depending on the pulse voltage and width. This helps to understand the tendency of pulse conditions and find the optimized stimuli for biological synaptic applications. The conductance change rate is defined as (*G*_final_ − *G*_initial_)/*G*_initial_. The conductance was extracted at a DC voltage of 0.5 V before and after the programming stimulus. The conductance varied by up to approximately 50 and 3.1 times for potentiation and depression, respectively. The rate of change in depression turned out to be relatively smaller than the rate of potentiation. This is because the reference value, that is, the denominator value, is the maximum conductance value that has undergone 50 potentiation procedures. The rate of conductance change is proportional to the stimulus intensity (pulse voltage) and the stimulus time (pulse width), as shown in Figure 4b,c. This can be associated with a phenomenon in which, if a human brain receives a stimulus having a large impact or a long stimulus, the memory can be retained for a relatively longer time than when exposed to a weak stimulus. The linear weight update is important for neuromorphic system applications, such as pattern recognition and voice recognition [48]. All potentiation and depression curves are presented as contour maps (Figure S2). Based on these potentiation data with four pulse width variations, the normalized conductance change was rearranged to compare nonlinearity (Figure 4d), which can be defined by the following equation [49], where the nonlinearity of an ideal case is 0:

(1) $$\mathrm{Nonlinearity}=\mathrm{Average}\left(\left|\frac{G_{device}-G_{ideal}}{G_{ideal}}\right|\right)\times 100\%$$

where *G_device_* is the measured normalized conductance value of the TiN/Ti/TiO_2_/SiO*_x_*/Si device and *G_ideal_* is the linear updated conductance value.

When a pulse width of 10 ms was applied to the device, the nonlinearity was 135.73%, which is its minimum value. Conversely, the nonlinearity reached 194.24%, its maximum value, when a pulse width of 100 ms was applied. Figure 4e shows the nonlinearity and dynamic range as functions of the pulse width. Note that the linearity degraded in spite of the fact that the dynamic range increased with the pulse width. Note also that the linearity improved with a decrease in the pulse width. This is because the change in the conductance was larger at the initial response when a longer pulse width was applied to the device.

**Figure 5:**
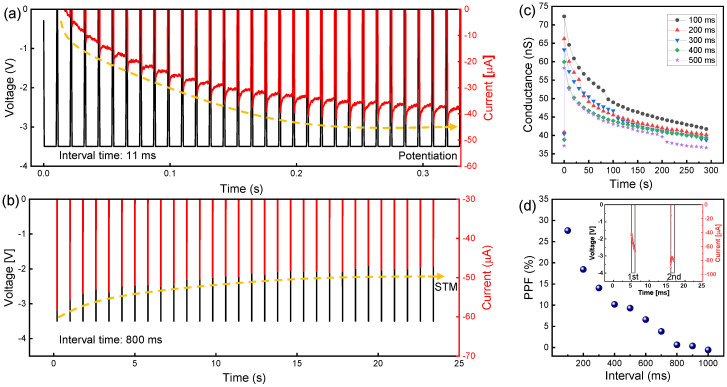
Short-term dynamics of TiN/Ti/TiO_2_/SiO*_x_*/Si device: (**a**) current was maintained by a short time interval (11 ms) after the set process; (**b**) current decayed from LRS in a long time interval (800 ms); (**c**) conductance decayed after 5 consecutive pulses were applied as a function of the time interval between pulses; (**d**) paired-pulse facilitation (PPF) as a function of interval time between paired pulses.

Another key biological synaptic function is STM. Short-term plasticity (STP) generated from the response of external momentary stimuli has a role in retaining the temporary information for filtering. To determine the feasibility of STP, we proceeded as follows: the current was varied through multiple pulse inputs at different frequencies; the current decay in terms of duration time and PPF were investigated, as shown in Figure 5. To increase the current for potentiation, an amplitude of −3.5 V, pulse widths of 10 ms, and a short time interval between pulses of 11 ms were applied (Figure 5a). By contrast, the current decayed slowly when an amplitude of −3.5 V, pulse widths of 10 ms, and a long time interval of 800 ms were applied (Figure 5b). This suggests that the proposed synaptic device can quickly and continuously store and process the input information. However, the information that comes into the stimulus with low frequency cannot retain the information. To determine how the stimulus applied at such an early stage could be retained and extinguished, the pulse interval-dependent current decay was measured, as shown in Figure 5c. A pulse amplitude of −4 V and a pulse width of 10 ms were applied, and the time interval between pulses was offset at 100 ms, from 100 to 500 ms. When five paired pulses were applied to the device with a similar initial conductance state and no stimulus, the shorter the interval, the greater the increase in conductance and the smaller the decay. This is because the device can retain more information in memory by providing additional stimulation before filtering the information. This suggests that the synapse temporarily strengthens the synaptic transmission when a neurotransmitter is introduced via a spike in the synapse. To quantify the enhancement, the current difference as a function of the paired-pulse interval condition was plotted, as shown in Figure 5d. Here, the PPF is defined as follows:

(2) $$\mathrm{PPF}=\;\left(\frac{I_{2nd}-I_{1st}}{I_{1st}}\right)\times 100\%$$

where *I*_1st_ and *I*_2nd_ are the currents of the first and second pulses, respectively, as shown in the inset of Figure 5d. When a stimulus is not offered for more than 1000 ms, as in the case of this PPF experiment, equilibrium is achieved; however, if the same stimulus is offered immediately after the initial stimulus, the synaptic transmission is enhanced.

**Figure 6:**
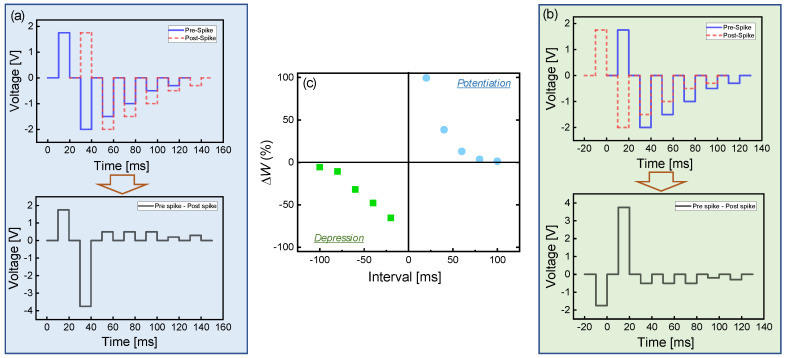
Spike-timing-dependent plasticity (STDP) of TiN/Ti/TiO_2_/SiO*_x_*/Si device: pulse schemes (pre- and postspike pulse trains) for (**a**) potentiaon and (**b**) depression; (**c**) STDP-like curve including potentiation and depression as a function of interval timing.

The adjacent neurons and synapse transmit signals using neurotransmitters electrically and chemically in which the synapse serves as a chemical exchange site for delivery from presynaptic neurons to postsynaptic neurons. STDP is a phenomenon in which the synaptic weight varies according to the temporal relationship between the stimulation of presynaptic and postsynaptic neurons. The connection of synapses can be either strong or weak depending on the timing of action potential firing between pre- and postsynaptic neurons. Figure 6a,b show a pulse train scheme that allows for the differentiation of voltage amplitude on every occasion. Prespike was fired before the postspike for potentiation (Figure 6a), and then later for depression (Figure 6b). The synaptic weight of the TiN/Ti/TiO_2_/SiO*_x_*/Si device was measured before and after applying two electric pulses (width: 10 ms), as shown in Figure 6c. The time difference between two spikes varied from −100 to +100 ms at intervals of 20 ms. When the prespike preceded the postspike, (Δ*t*_(pre-post)_ > 0), the effective pulse amplitude increased for potentiation and then the synaptic weight was increased. As the time delay increased, the effective amplitude of the voltage decreased, which confirms that the amount of weight change was reduced. Conversely, when the postspike precedes the prespike, (Δ*t*__(pre-post)_ < 0), the depression phenomenon occurred. The STDP behavior in our device (Figure 6c) was similar to the asymmetric Hebbian learning rule phenomenon, which is one of the ideal STDP functions used in computational models [50].

**Figure 7:**
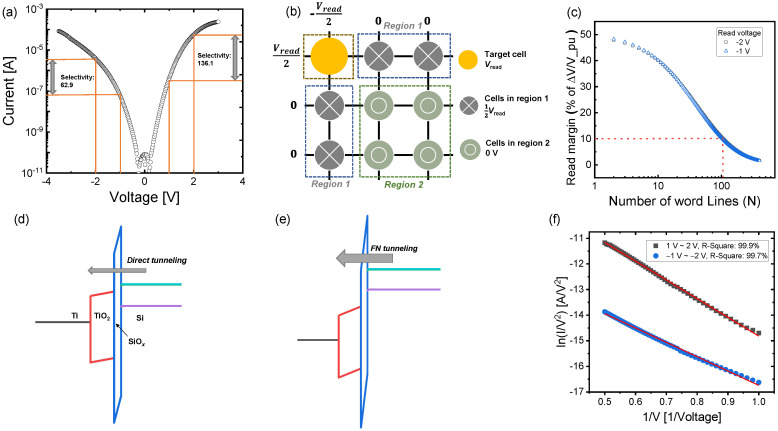
Nonlinear characteristics of TiN/Ti/TiO_2_/SiO*_x_*/Si device: (**a**) I–V curve with high selectivity in LRS pulse schemes; (**b**) half-bias scheme in cross-point array structure; (**c**) read margin as a function of number of word lines; energy band diagrams at (**d**) low voltage and (**e**) high voltage; (**f**) ln(I/V^2^) versus 1/V plot for Fowler–Nordheim (FN) tunnel fitting.

Next, we investigated the nonlinear I–V characteristics of the TiN/Ti/TiO_2_/SiO*_x_*/Si device for a high-density synaptic device array. Figure 7a shows the I–V curve in an LRS. Selectivity is defined as the ratio between the current at the read voltage (*V_read_*) and the current at half of *V_read_*. The selectivities at *V_read_* of 1 and −1 V were 136.1 and 62.9, respectively. The high nonlinearity of the I–V curve in LRS can minimize the sneak current in the cross-point array. The sneak current can dominantly flow through the adjacent cells with a low resistance (especially the cells in the LRS). The half-bias read-margin scheme was applied to the cross-point array structure in Figure 7b—0.5*V_read_* and zero voltage at the cells in region 1 and the cells in region 2 were applied, respectively, while *V_read_* was applied to the target cell. The highly nonlinear behavior of the TiN/Ti/TiO_2_/SiO*_x_*/Si device indicates that the read current at 0.5 *V_read_* in the LRS can be suppressed. The read margin as a function of the number of word lines (*N*) is calculated using the following expression:

(3) $$\frac{R_{pu}}{\left(\left[R_{LRS}\left(V_{read}\right)\right]\;\|\;\left[\frac{2R_{LRS}\left(\frac{V_{read}}{2}\right)}{\left(N-1\right)}\right]\right)+R_{pu}}-\;\frac{R_{pu}}{\left(\left[R_{HRS}\left(V_{read}\right)\right]\;\|\;\left[\frac{2R_{LRS}\left(\frac{V_{read}}{2}\right)}{\left(N-1\right)}\right]\right)+R_{pu}}$$

where *R_pu_* is the pull-up resistance that is connected to the equivalent circuit for the cells in the cross-point array [51]. The read margin decreases with the array size because the sneak current path increases. The number of word lines was greater than 100 to secure a read margin of 10 when the *V_read_* was −1 and −2 V (Figure 7c). The plausible mechanism of nonlinear I–V characteristics could be explained by direct tunneling and Fowler–Nordheim (FN) tunneling [52] at a low voltage and high voltage, respectively. FN tunneling is expressed as follows:

(4) $$J_{FN}=\frac{{\left(qE\right)}^{2}}{8\pi h\emptyset_{B}}\exp\left[\frac{-8\pi \sqrt{2qm^{*}}}{3hE}\right]\emptyset_{B}{}^{3/2}$$

where *q* is the electronic charge, *E* is the electric field, *h* is the Planck constant, m* is the effective electron mass, and $\emptyset_{B}$ is the energy barrier that is overcome by the electron.

The SiO*_x_* layer with a higher band gap on the Si substrate acts as a tunnel barrier role. A voltage-dependent carrier injection results in highly nonlinear characteristics. Direct tunneling allows a very low current given that the carriers pass through the intact SiO*_x_* thickness (Figure 7d). By contrast, a triangular barrier at a high voltage has the effect of reducing the effective tunneling thickness for the carriers (Figure 7e). The I–V curves of high-voltage regions (1~2 V and −1~−2 V) in an LRS are well fitted with the ln(I/V^2^) versus the 1/V plot. This confirms the underlying FN tunneling mechanism of the TiN/Ti/TiO_2_/SiO*_x_*/Si device (Figure 7f). In FN tunnel fitting, the I–V curves in the LRS fit well from approximately 1 V (fitting accuracy, R-square, is more than 99%). The initial voltage at FN tunnel fitting (1 V) is defined as the critical voltage. Considering the dielectric constants of two dielectric materials (TiO_2_: ~80 and SiO_2_: ~4) [53,54], most of the voltage could be applied to the SiO*_x_* layer according to Gauss’s law. Therefore, it can be assumed that the critical electric field is approximately 4.76 MV/cm when a critical voltage of 1 V for FN tunneling is applied to the 2 nm thick SiO*_x_* layer. The critical electric field in the TiN/Ti/TiO_2_/SiO*_x_*/Si device system is slightly smaller than the values (6 to 8 MV/cm) reported in previous study [55]. This is because some defects are induced in the SiO*_x_* layer in an LRS.

Finally, we surveyed the TiO_x_-based RRAM devices that were previously reported in Table 1 [39,40,56,57,58,59,60,61]. TiO_2_ dielectrics as RRAM devices were prepared by various methods such as radio frequency (RF) sputtering, DC sputtering, atomic layer deposition (ALD), epitaxy, and spin coating. Both the filamentary and interface types, as two of the typical RRAM switching, were observed. For the filamentary type, the LRS current hardly changes depending on the active area of the device [57]. Conversely, in the case of the interface type, it is commonly observed that the LRS current decreases as the area of the device decreases [40,56]. Additionally, there are more and more reports on neuromorphic applications using the advantage of multiconductance of interface type switching [39,56].

## 4. Conclusions

In summary, the set and reset processes in TiN/Ti/TiO_2_/SiO*_x_*/Si synaptic devices occur gradually, making it suitable for the imitation of biological synapses and STP functions. The synaptic plasticity of the proposed device was well controlled under various input pulse amplitudes and widths. The larger these two parameters, the greater the amount of conductance change, which means that a stimulus having a larger impact or an impact for a long time can control the persistence of the memory state. Short-term plasticity, such as PPF, is controllable using different time intervals. In addition, the synaptic weight with firing time difference is controlled through the proposed pulse schematic for STDP. Finally, highly nonlinear I–V curves in an LRS originating from the SiO*_x_* tunnel barrier are beneficial for high-density synapse arrays. The proposed synaptic device shows potential to become a basic building block in hardware neuromorphic systems by obtaining multiple conductance modulations.

## Figures and Tables

**Table 1:** Comparison of TiO_x_-based Resistive random access memory (RRAM) devices prepared by different techniques.

Device Structure	Dielectric Deposition Method	Dielectric Thickness	Operation Voltage	Operation Current	Switching Type	Applications
Mo/TiO_x_/TiN [39]	RF sputtering	15 nm	Set: 3 V Reset: −3 V	<1 μA	Interface	Non-volatile memory Neuromorphic
Ti/TiO_2−x_/TiO_2−y_/Au [40]	RF magnetron sputtering	45 nm	Set: 6 V Reset: −5 V	<100 μA	Interface	Non-volatile memory
Pt/TiO_2−x_/TiO_2_/Pt [56]	Atomic layer deposition	12 nm	Set: 2 V Reset: −2 V	<1 mA	Interface	Non-volatile memory Neuromorphic
Ti/TiO_2_/Nb-SrTiO_3_ [57]	Epitaxy	10 nm	Set: 2.5 V Reset: −1 V	<10 mA	Filamentary	Non-volatile memory
Pt/TiO_2−x_/Pt [58]	Reactive sputtering	5 nm	Set: 4 V Reset: −3.6 V	<1 mA	Interface	Non-volatile memory
Pt/TiO_2_/Pt [59]	Atomic layer deposition	15 nm	Set: −2 V Reset: < 2 V	<4 mA	Filamentary	Non-volatile memory
Pt/TiO_x_/Pt [60]	Plasma enhanced atomic layer deposition	7 nm	Set: 3 V Reset: 2.25 V	>1 mA	Filamentary	Non-volatile memory
Pt/TiO_2_/W [61]	Sol-gel spin coating	>100 nm	Set: 1.25 V Reset: −1.25 V	>10 μA	Interface	Non-volatile memory
Ti/TiO_2_/SiO*_x_*/Si [This work]	Reactive sputtering	13 nm	Set: −3.5 V Reset: 4 V	<400 μA	Interface	Non-volatile memory Neuromorphic

## Supplementary Materials

The following are available online at https://www.mdpi.com/2079-4991/10/9/1821/s1, Figure S1: Statistical distribution (cycle-to-cycle) of TiN/Ti/TiO2/SiOx/Si device in the HRS and LRS, Figure S2: Potentiation and depression curves of TiN/Ti/TiO2/SiOx/Si device depending on the pulse width. Potentiation: (a) 100 μs, (b) 1 ms, (c) 10 ms, (d) 100 ms. Depression: (e) 100 μs, (f) 1 ms, (g) 10 ms, (h) 100 ms.
