# Supplementary material for: Short-Term Memory Dynamics of TiN/Ti/TiO2/SiOx/Si Resistive Random Access Memory
Source: Nanomaterials (Basel). 2020 Sep 12;10(9):1821. doi: 10.3390/nano10091821 (PMC7559005; doi:10.3390/nano10091821)
Supplement: Supplementary file 1 [file nanomaterials-10-01821-s001.pdf]

Supplementary Materials

Short-Term Memory Dynamics of $TiN/Ti/TiO_2/SiO_x/Si$ Resistive Random Access Memory

Hyojong Cho and Sungjun Kim *

Division of Electronics and Electrical Engineering, Dongguk University, Seoul 04620, South Korea; chj9102@dgu.ac.kr

* Correspondence: sungjun@dongguk.edu

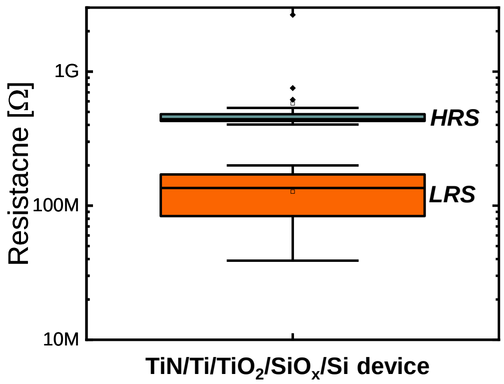

Figure S1. Statistical distribution (cycle-to-cycle) of $TiN/Ti/TiO_2/SiO_x/Si$ device in the HRS and LRS.

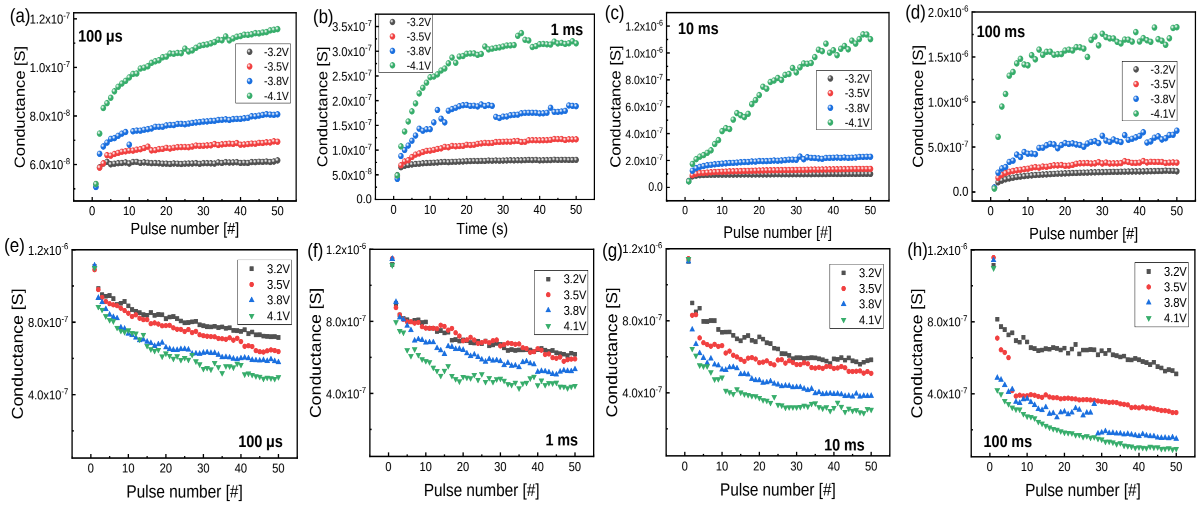

Figure S2 Potentiation and depression curves of TiN/Ti/TiO_2/SiO_x/Si device depending on the pulse width. Potentiation: (**a**) 100 μs, (**b**) 1 ms, (**c**) 10 ms, (**d**) 100 ms. Depression: (**e**) 100 μs, (**f**) 1 ms, (**g**) 10 ms, (**h**) 100 ms.
